# A widely distributed genus of soil Acidobacteria genomically enriched in biosynthetic gene clusters

**DOI:** 10.1038/s43705-022-00140-5

**Published:** 2022-08-13

**Authors:** Alexander Crits-Christoph, Spencer Diamond, Basem Al-Shayeb, Luis Valentin-Alvarado, Jillian F. Banfield

**Affiliations:** 1 Department of Plant and Microbial Biology, University of California, Berkeley, CA USA; 2 Department of Earth and Planetary Science, University of California, Berkeley, CA USA; 3 Department of Environmental Science, Policy, and Management, University of California, Berkeley, CA USA; 4 Earth Sciences Division, Lawrence Berkeley National Laboratory, Berkeley, CA USA; 5 Chan Zuckerberg Biohub, San Francisco, CA USA

**Keywords:** Soil microbiology, Metagenomics

## Abstract

Bacteria of the phylum Acidobacteria are one of the most abundant groups across soil ecosystems, yet they are represented by comparatively few sequenced genomes, leaving gaps in our understanding of their metabolic diversity. Recently, genomes of Acidobacteria species with unusually large repertoires of biosynthetic gene clusters (BGCs) were reconstructed from grassland soil metagenomes, but the degree to which species with this trait are widespread is still unknown. To investigate this, we assembled 46 metagenome-assembled genomes recovered from permanently saturated organic-rich soils of a vernal (spring) pool ecosystem in Northern California. We obtained high and medium-quality draft genomes for three novel species from *Candidatus* Angelobacter (a proposed subdivision 1 Acidobacterial genus), a genus that is genomically enriched in genes for specialized metabolite biosynthesis. Acidobacteria were particularly abundant in the vernal pool sediments, and a *Ca*. Angelobacter species was the most abundant bacterial species detected in some samples. We identified numerous diverse biosynthetic gene clusters in these genomes, and also in five additional genomes from other publicly available soil metagenomes for other related *Ca*. Angelobacter species. Metabolic analysis indicates that *Ca*. Angelobacter likely are aerobes that ferment organic carbon, with potential to contribute to carbon compound turnover in soils. Using metatranscriptomics, we identified *in situ* metabolic activity and expression of specialized metabolic traits for two species from this genus. In conclusion, we expand genomic sampling of the uncultivated *Ca*. Angelobacter, and show that they represent common and sometimes highly abundant members of dry and saturated soil communities, with a high degree of capacity for synthesis of diverse specialized metabolites.

## Introduction

It is estimated that an overwhelming majority of soil bacterial species have thus far been recalcitrant to cultivation [1], and these uncultivated bacteria are not evenly distributed across the tree of life [2]. While many phyla of bacteria found in soils have few cultivated representatives, there are few as ubiquitous and diverse as the Acidobacteria [3]. From metagenomic and 16S rRNA surveys we have learned that Acidobacteria are collectively the most abundant phylum in soils [4], harboring significant taxonomic diversity [5, 6], with over 26 accepted subdivisions [7]. However, they are relatively undersampled in cultivation efforts, with fewer than 100 sequenced isolate genomes from the entire phylum deposited into the RefSeq database as of the start of 2021. Some reported isolates have also not been genomically sequenced or deposited into public strain collections, complicating the study of even previously cultivated members of the phylum. Isolate-based studies of soil Acidobacteria indicate that they are often heterotrophic, aerobic, and capable of complex carbon degradation, and are thought to be mostly oligotrophic [3]. However, it is unclear to what degree these findings extrapolate to the diversity of the entire phylum in soils.

More recently, genome-resolved metagenomics, or the process of assembling and curating genomes directly from metagenomes, has been applied to soil bacterial communities and resulted in the assembly of hundreds of novel soil Acidobacteria genomes [8–10]. Genome annotation and functional prediction from these genomes have improved our understanding of the phylum’s metabolic potential. Metabolic characteristics predicted from genomes of uncultivated Acidobacteria include the ability to degrade many complex carbohydrate compounds via carbohydrate active enzymes, the capacity for nitric oxide reduction, and the ability to oxidize methanol [8]. It also was previously shown that multiple Acidobacteria genomes from metagenomic data from a single soil ecosystem encode numerous gene clusters for the biosynthesis of specialized metabolites [11]. In particular, two lineages of Acidobacteria in subgroups 1 and 4, designated *Candidatus* Angelobacter (Genome Taxonomy Database genus g__Gp1-AA17; NCBI taxon “Acidobacteria bacterium AA117”) and *Candidatus* Eelbacter (NCBI taxonomy Blastocatellia bacterium AA13), respectively, were found to possess large repertoires of biosynthetic genes. Specifically, these genomes each encoded for 300–400 Kb of nonribosomal peptides synthetases (NRPSs) and polyketides synthases (PKSs). A third lineage of uncultivated Acidobacteria has also been reported which also possessed similar numbers of NRPS and PKS gene clusters, and was sequenced from ocean biofilm samples [12]. Additional genomes from this third lineage have since been noted for their unusually large nonribosomal peptide genes [13]. However, from the genomes reported thus far, it remains unclear how these three lineages are related, or whether they are widespread in soil environments. Here, we extend these results using publicly available Acidobacteria genomes from a variety of soil types from the Genome Taxonomy Database, genomes assembled from metagenomes in the IMG database, along with sampling and new metagenomic analysis of saturated soils from a vernal pool ecosystem. Of the previously reported Acidobacteria lineages enriched in biosynthetic gene clusters, we identified several more species from *Ca*. Angelobacter, and focused our analysis on this group. Our results include eight genomes from the *Ca*. Angelobacter genus in addition to the single previously reported genome, and suggest that a significant investment in secondary metabolism is a common feature of soil bacteria from this lineage.

## Methods

### Field sampling

We collected 29 soil samples from a seasonal vernal pool in Lake County, California (near coordinates 38.686N 122.525W) in October 2018 and October 2019. In the first year, we collected four samples from a soil depth of 20–30 cm, 4 from 30 to 50 cm, and 2 from 60 to 80 cm. In the following year, we collected 9 samples from 20 to 30 cm, 8 from 30 to 60 cm, and 2 from 60 to 100 cm (Supplementary Table S1). Samples were either stored on dry ice for DNA extraction or flash-frozen in ethanol cooled with dry ice for RNA extraction. Samples were collected evenly across directly adjacent transects that were under 5 m in length. Samples for RNA extraction were obtained during the 2019 sampling year, and were collected in tandem DNA samples across the transects. The Qiagen PowerSoil Max DNA extraction kit was used to extract DNA from 10 g of soil, and the Qiagen AllPrep DNA/RNA extraction kit was used to extract RNA from 2 g of soil, in line with recommended input quantities for each kit. Samples were sequenced by the QB3 sequencing facility at the University of California, Berkeley on a NovaSeq 6000. Read lengths for the 2018 DNA samples and the RNA samples were 2 × 150 bp, and then 2 × 250 bp sequencing was used for the 2019 DNA samples to assist with assembling higher quality genomes. A sequencing depth of 10 Gb was targeted for each of the 2018 samples, and 20 Gbp for each of the 2019 samples. Geochemical measurements (concentrations of Total Carbon, Total Nitrogen, Calcium, Zinc, Magnesium, Copper, and Iron) were performed on ten selected soil samples from the sampling site at the University of California, Davis Analytical Laboratory (https://anlab.ucdavis.edu/methods-of-analysis) using publicly available protocols.

### Metagenomic assembly and annotation

Metagenomic sequencing reads were assembled separately for each sample using the IDBA_UD assembler [14]. Contigs greater than 2.5 Kb were retained for binning and sequencing reads from all samples were cross-mapped against each sample’s assembly using Bowtie2 [15]. The resulting differential coverage profiles were filtered at a 95% read identity cutoff, and then used for genome binning with MetaBAT2 [16]. Resulting genome bins were assessed for completeness and contamination using CheckM [17]. Abnormal (>75% of hits to non-Acidobacteria species) taxonomic distributions of top BLASTP hits for predicted proteins on contigs were used to remove likely contaminating contigs from genomes with GGKBase. Taxonomy was assigned to genome bins and a phylogenetic tree was constructed using phylogenetic placement of single copy marker genes with GTDB-Tk [18]. *Candidatus* Angelobacter genomes were identified by classification with GTDB-Tk as either family “Gp1-AA117” or genus “Gp1-AA17”; nomenclature derived from the first identified genome Angelobacter Gp1-AA117. Community relative abundance profiles were determined for each sample using the GraftM [19] metagenomic classifier and the ribosomal protein L6 marker gene. Publicly available assembled metagenomic datasets were searched using the Integrated Microbial Genomes (IMG) web portal’s “Metagenomic bins by phylogenetic category” function, and 243 Acidobacteria genome bins were downloaded from 224 soil metagenomes (Supplementary Table S2). Genomes were placed in a concatenated ribosomal protein phylogeny with GTDB-Tk (pplacer) and five additional close relatives of the initial *Candidatus* Angelobacter genome were identified (Supplementary Table S5).

Genes were predicted on all genomes using Prodigal with default settings [20]. Biosynthetic gene clusters were annotated in genomes using antiSMASH 5.0 [21]. The number of Condensation domains and Ketoacyl synthase domains was determined by using HMMER3 [22] and querying all antiSMASH predicted biosynthetic proteins using the PF00109 and PF00668 Pfam HMMs. BiG-SCAPE [23] was used to generate gene cluster families of BGCs, with a clustering cutoff of 0.3 and a global alignment mode. Comparisons to the Minimum Information about a Biosynthetic Gene cluster (MIBiG) database of characterized BGCs were also performed by passing the –mibig parameter to BiG-SCAPE. KEGG functional annotations for genes across the entire genomes were obtained using METABOLIC [24]. Metatranscriptomic sequencing reads were mapped to all genome bins using Bowtie2, and filtered to only paired-end reads with >95% identity using a custom Python script. Read counts per transcript were then normalized by total number of sampling reads and length of each transcript, and RPKM per gene was calculated using the formula RPKM = Read count * (1/(gene length/1000)) * (1/(total reads/1,000,000)) using a custom Python script.

## Results

### Sampling and metagenomic sequencing

**Fig. 1:**
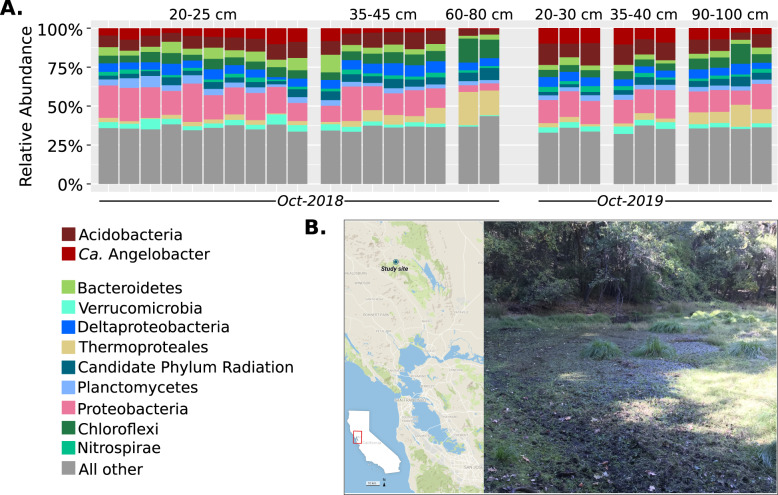
Community composition of vernal pool soils. **A** Ribosomal protein (L6) abundances and taxonomic classifications across all metagenomic samples obtained in this study. The abundances of *Ca*. Angelobacter (*Gp1-AA117)* are shown separately from all other hits in phylum Acidobacteria. **B** Photograph of the vernal pool that was metagenomically sampled in this study, in Lake County, California, USA.

We sequenced 29 soil metagenomes from a seasonal vernal pool in Lake County, California, USA in October 2018 and October 2019 (Supplementary Table S1). The elevation of the site is ~600 m and the pool is surrounded by Douglas Fir and Oak (Fig. 1B). The soils at the site are fine-grained, organic-rich mud, and clay-rich at depth, surrounded by gravelly loam Inceptisols formed in material weathered from rhyolitic tuff. Sampling occurred when the pool was at its driest before the first major autumn rainfall, along a transect in the pool bed that would be covered by water for a majority of the year. Total nitrogen and total carbon measured at the site averaged 1% and 13% respectively, both decreased with increasing soil depth (Supplementary Table S3). The soils were slightly less carbon rich at depth, where by 80 cm total carbon decreased to <10%. All samples were saturated with water at the time of collection. Metagenomic assembly resulted in on average 232 Mb of assembled sequence in contigs >2.5 Kb per sample. Using these assemblies, we generated 46 dereplicated Acidobacterial genomes of at least medium-quality (>90% complete, <10% contaminated) (Supplementary Table S4).

### Community composition and assembled genomes

In order to assess the bacterial community composition of the vernal pool, the L6 ribosomal protein was used as a phylogenetic marker with GraftM [19]. The bacterial communities at the site were found to be less complex than the soil communities observed by a previous metagenomic effort in an arid grassland meadow [8]. Dominant taxa included species in the phylum Acidobacteria, Proteobacteria, Chloroflexi, with a variety of Archaea dominating the community at deeper depths of 60–80 cm (Fig. 1A). At high taxonomic ranks of bacteria and archaea, community composition was fairly consistent within each stratum across depths. Members of the Candidate phyla radiation consistently composed ~10% of the community, more than has been previously reported in drier soils [25].

**Fig. 2:**
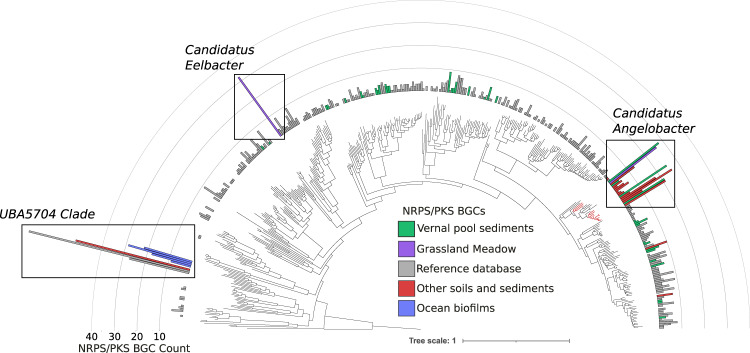
Phylogeny of the Acidobacteria annotated with number of NRPS and PKS BGCs per genome. Concatenated ribosomal protein phylogeny of all Acidobacteria genomes in NCBI GenBank, additional genomes obtained from IMG, and genomes obtained from this study (“Vernal pool sediments”). Plotted is the number of BGCs annotated as NRPS and PKS per genome, and genomes are colored by their ecosystem of origin.

Of particular interest was the high abundance of the phylum Acidobacteria within the vernal pool soil microbial community, in some samples reaching 25% relative abundance. The 46 assembled near-complete species-dereplicated Acidobacteria genomes from the site were placed in a phylogenetic tree of all 370 known Acidobacteria species in the NCBI Assembly database and the Genome Taxonomy Database (GTDB) using a concatenated set of ribosomal proteins (Fig. 2). Genomes recovered from the vernal pools samples derived from five different Acidobacteria classes (Supplementary Table S3), indicating a wide diversity of species abundant for this phylum in the vernal pool.

Phylogenetic analysis of single-copy marker genes identified three novel genomes related to the uncultivated group 1 Acidobacteria, *Ca*. Angelobacter (GTDB genus g__Gp1-AA17; NCBI taxon “Acidobacteria bacterium AA117”). Besides the previously published genome from another site in Northern California, there was only one other preexisting metagenome-assembled genome from this clade, recovered from a thawing permafrost peatland located in arctic Sweden. The three new genomes obtained from the vernal pool study site were all near-complete with low estimated contamination and ranged in size from 6 to 7 Mb, with an average GC content of 55% (Supplementary Table S5). One of these genomes, *SRVP-Angelobacter-2*, was 6.44 Mb total across 42 contigs, which represents the most contiguous assembly of any *Candidatus Angelobacter* species to date, and significantly more contiguous than the previously published genome. Assembly of contiguous genome fragments is important for accurate binning and especially for the recovery of complete BGCs and identification of nearby genomic features. *Angelobacter* were at reasonably high abundances in all samples, and the *SRVP-Angelobacter-3* species was the most abundant organism in samples from 20 cm depth. Two of the genomes, *SRVP-Angelobacter-2* and *SRVP-Angelobacter-3*, contained 16S rRNA genes with 96.8% sequence identity, consistent with being members of the same genus.

To further expand our characterization of the *Ca*. Angelobacter genus, we searched the IMG database of assembled metagenome bins for additional metagenome-assembled genomes from the genus by phylogenetic placement of all acidobacterial genomes in the dataset. We identified additional draft genomes for five more species in the *Ca*. Angelobacter, all from soils. Three were from a deeply sequenced metagenomic study of corn and switchgrass rhizosphere in Michigan [26], one from a metagenomic study of soils amended with Pyrogenic organic matter in New York [27], and one genome was obtained from a mini metagenomic selection approach from Massachusetts forest soils [28] (Supplementary Table S5). Comparing these genomes by average nucleotide identity (ANI), we found that six of the Angleobacter genomes clustered together with >60% ANI (Fig S1). The genome obtained from the New York study intriguingly shared 96.8% ANI with one of the genomes obtained from Michigan soils, indicating that they could be considered the same species based on a 95% ANI definition of microbial species [29]; all of the other genomes appeared to be separate species.

### Many species of *Candidatus* Angelobacter genus possess diverse biosynthetic gene clusters

**Fig. 3:**
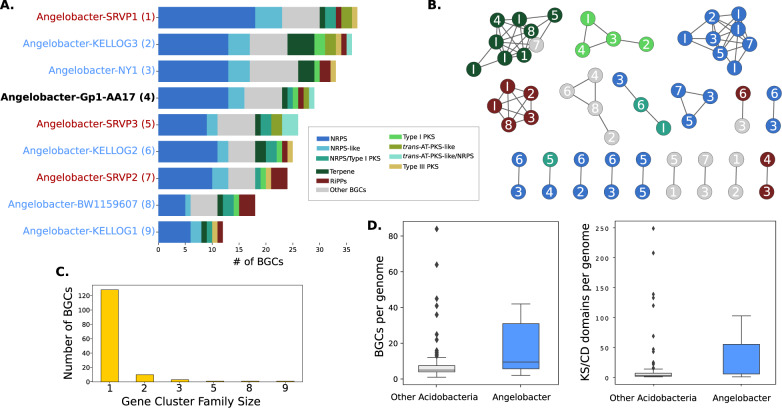
Biosynthetic gene clusters from genomes in *Candidatus* Angelobacter. **A** The number and class of BGCs in each species genome from *Ca*. Angelobacter with at least 10 BGCs. The previously published reference genome for this genus is bolded in contrast to new genomes obtained in this study (red) and genomes identified in other studies (blue). **B** A BiG-SCAPE gene cluster family network of BGCs from *Ca*. Angelobacter. Each node is a BGC, connected to other similar BGCs by genomic similarity. Singleton BGCs are not shown. BGCs are numbered by their corresponding genome of origin, as denoted in part (a). BGCs from incomplete *Ca*. Angelobacter genomes not included in part (a) are marked with ‘I’. Nomenclature: C, Condensation Domain; Ser, Adenylation Domain (Serine); The, Adenylation Domain (Threonine); T, Peptidyl Carrier Protein; Phe, Adenylation Domain (Phenylalanine). E, Epimerase. **C** The number of *Ca*. Angelobacter BGCs in gene cluster families of each size. **D** The number of BGC s per genome and ketoacyl synthase and NRP condensation domains per genome for all genomes from *Ca*. Angelobacter compared to the rest of the Acidobacteria.

The previous genome reported from *Ca*. Angelobacter (accession GCA_003223515) was reported as notable in its substantial genomic capacity for production of specialized metabolites (14.9% of the genome), particularly via biosynthetic gene clusters of nonribosomal peptide synthetases and polyketide synthases [11]. To understand how consistent this trait is across this genus, we ran antiSMASH 5.0 on all of the recovered genomes to identify Biosynthetic Gene Clusters (BGCs) and predicted both polyketide keto-synthase (KS) and NRPS condensation (CD) protein domains across antiSMASH BGCs. Visualizing the number of these biosynthetic domains found per genome, it is clear that the *Ca*. Angelobacter genus stands out in the acidobacterial phylum (Figs. 2, 3D), and contains both significantly more BGCs and KS/CD domains than the average Acidobacteria (Mann-Whitney *t* test; *p* < 0.001). We also note the existence of two other independent acidobacterial clades with unusual numbers of biosynthetic enzymatic domains. The first, *Candidatus Eelbacter*, is a group 4 Acidobacteria genome previously reported [11], and the second is a lineage represented by genomes previously reported from ocean biofilm and soil metagenomes that cluster phylogenetically with each other and are identified by similarity to a genome deposited with the moniker “UBA5704” [30]. Within the *Ca*. Angelobacter clade, we also identified a minority of genomes with few biosynthetic gene clusters, indicating that genomically encoded biosynthetic capacity may vary within this lineage. Percentage of the genome dedicated to secondary metabolism ranged from 1.8% to 11.2%, with a median value of 9.7% for the *Ca*. Angelobacter genomes included in this analysis (Supplementary Table S5). This variability can also be the case for some members of the Actinomycetales, which are renowned for specialized metabolite production in general, yet the trait can be patchy across individual species [31]. There is a clear phylogenetic distribution of the genomes with fewer BGCs clustering together within the larger clade (Fig. 2), but the possibility also remains that BGCs were not assembled or binned properly in some *Ca*. Angelobacter genomes from metagenomes.

The total number of BGCs in newly recovered *Ca*. Angelobacter genomes in many cases rivals or outnumbers the previously reported *Ca*. Angelobacter genome’s biosynthetic gene content (Fig. 3A; Supplementary Table S6). Many *Ca*. Angelobacter BGCs were NRPS or NRPS-PKS hybrids over 100 Kb in length, and NRPS genes within the clusters were often large, with the largest ORF in the genus being 24 Kbp in length. While direct functional prediction of highly novel BGCs is challenging from genomic data, we noted that no biosynthetic gene cluster contained adjacent known genomic markers of siderophore biosynthesis (such as TonB-dependent receptors or periplasmic binding proteins), but were often associated with adjacent genes for MacB tripartite efflux pumps. We identified 61 genes for MacB efflux pumps within *Ca*. Angelobacter BGCs, which made this transporter more frequently associated with *Ca*. Angelobacter BGCs than all other identifiable transporter genes combined.

To clarify whether *Ca*. Angelobacter species tend to share similar BGCs, we applied the BiG-SCAPE workflow to the recovered *Ca*. Angelobacter BGC collection to identify gene cluster families, or groups of related BGCs. We found that the majority of BGCs in the collection were singletons, indicating substantial genetic diversity and comparatively few BGCs shared between species (Fig. 3B, C). Of BGC families that were shared by species, the majority were only shared by two species; only five clusters were shared amongst more than three *Ca*. Angelobacter species. The gene cluster families that were commonly shared include terpene, Type I PKS, NRPS, and a ribosomally synthesized peptide gene cluster family. While most of the shared gene cluster families were short BGCs, two *Ca*. Angelobacter species from different study sites shared a multi-domain NRPS cluster with near-identical adenylation domain structure (Supplementary Table S6). No *Ca*. Angelobacter BGCs were similar enough in gene content to any characterized BGCs in the MIBiG database to be linked in the resulting BiG-SCAPE network.

### Primary metabolic features of *Candidatus* Angelobacter species

Genomic inferences about primary metabolisms can help inform understanding of a microorganisms’ lifestyle and trophic niche, while also possibly guiding cultivation efforts. Generally the predicted metabolic capabilities of the three *Candidatus* Angelobacter genomes recovered from vernal pool sediments with more than 10 NRPS/PKS BGCs (SRVP1, SRVP2, and SRVP3) identify them as aerobic heterotrophs with reasonable capacity for complex carbohydrate degradation and assimilation (Supplementary Tables S7, S8). We examined genomic potential for specific metabolic traits that allow for plasticity in carbon assimilation and energy generation across these *Ca*. Angelobacter genomes. *Ca*. Angelobacter have complete pathways for glycolysis and oxidative pentose phosphate conversions (Supplementary Tables S6, S7). They have identifiable homologs of all enzymes in the TCA cycle, with the exception of fumarate hydratase (Supplementary Tables S6, S7). Unless this gene is missing from all three assemblies, or this function is performed by an unidentified homolog, it is more likely that in these organisms the TCA reactions act as a source of metabolic intermediates than a major source of reducing power. They also each encode a four complex oxygenic respiratory chain including an NADH:quinone oxidoreductase (Complex I), succinate dehydrogenase (Complex II), cytochrome b containing Complex III, and oxygen utilizing cytochrome c oxidase (Complex IV). *Ca*. Angelobacter SRVP1 and SRVP2 also encode a separate oxygen-utilizing cytochrome bd-like ubiquinol oxidase, which is thought to operate under lower oxygen availability [32]. Two putative mechanisms for anaerobic energy generation are also present: fermentation to ethanol and the ability to reduce nitrate to nitrite (Supplementary Tables S7, S8). Given that these organisms encode a full set of respiratory complex enzymes, with oxygen utilizing terminal oxidase, they are likely primarily aerobics, but with possible facultative anaerobic metabolism. They also have the ability to assimilate acetate (and other 2 carbon compounds) into biomass by encoding enzymes for the glyoxylate shunt, which can provide additional metabolic flexibility for situations where complex carbohydrates or hexose sugars are unavailable. They encode for multiple routes for producing the precursors for polyketide biosynthesis (acetyl-CoA, propionyl-CoA, and malonyl-CoA) including the capability to import, and degrade branched-chain amino acids to these precursors (Supplementary Table. S8). Finally, one of the *Ca*. Angelobacter genomes (*SRVP-Angelobacter-2*) from the vernal pool soil contained a Type IC CRISPR-Cas array and another (*SRVP-Angelobacter-3*) contained a Type IIID CRISPR-Cas system, indicating some degree of pressure from phage predation for these species.

### *Candidatus* Angelobacter species are transcriptionally active in situ

**Fig. 4:**
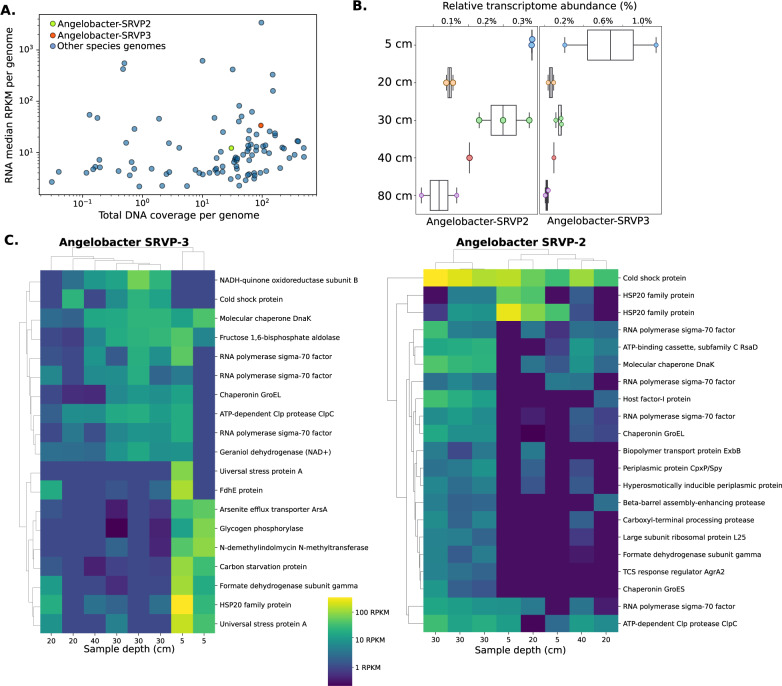
Metatranscriptomic activity of *Candidatus* Angelobacter species in situ. **A** RPKM-normalized metatranscriptomic (*y*-axis) and coverage-normalized metagenomic (*x*-axis) reads mapping to two *Ca*. Angelobacter species, compared to all other microbes with genomes obtained from the vernal pool site, in samples from soil depths of 5–20 cm. **B** Genome-wide transcriptional activity for two *Ca*. Angelobacter species, compared by sediment depth of sampling. **C** Transcriptomic RPKM of the 50 most highly expressed genes with assigned Kegg Orthologs (KOs) at each sampling depth for the two *Ca*. Angelobacter species.

To track transcriptional activity of *Ca*. Angelobacter in situ, we flash-froze soil samples in the field to preserve for metatranscriptomic RNA extraction, extracting and sequencing 20 Gbp of RNA for ten samples taken from the vernal pool study site in 2019. Mapping both DNA and RNA reads back to genomes obtained from the site, we were able to track both relative abundance (DNA) and relative transcriptional activity (RNA) for microbes of interest. Two *Ca*. Angelobacter species (*SRVP-2* and *SRVP-3*) were found to be more transcriptionally active than 63% and 84% (respectively) of other bacteria with genomes obtained from the site at soil depths of 5–20 cm (Fig. 4A), while only being 43% and 79% (respectively) more abundant in DNA samples, indicating an above-average relative transcriptional activity when compared to other bacterial species at the site. Another genome, *SRVP-Angelobacter-1*, was observed to have only very low transcriptional activity in the metatranscriptomes.

Overall, for the two active *Ca*. Angelobacter species (SRVP-2 and SRVP-3) we identified transcripts for 20% and 22% of their genes, respectively. The low median level of detectable transcriptional activity reflects the complexity of soil metatranscriptomes. We compared total expression for these two *Ca*. Angelobacter species by soil depth of the sample, and found the highest relative levels of transcriptional activity in our 5 cm depth samples, followed by the 30 cm depth samples, and the lowest levels of activity in the 80 cm deeper samples (Fig. 4B).

We next identified and annotated the 50 most abundant transcripts from either *Ca*. Angelobacter species in the dataset at both shallow (5–20 cm) and deeper depths (30–60 cm) (Fig. 4C). The transcriptional activity of highly expressed genes did not perfectly cluster by soil depth across samples. Intriguingly, we observed a unique transcriptomic profile for *SRVP-Angelobacter-3* in two samples taken from a 5 cm depth, in which several stress-related genes were highly expressed. The three samples collected at a depth of 30 cm clustered together by transcriptomic activity for both species, and were characterized by high expression for a set of genes that seemed to be involved in growth and general metabolism: ribosomal proteins, formate dehydrogenase, and a chromosomal segregation protein (Fig. 4C). We observed expression for about 5% of genes found in antiSMASH biosynthetic gene clusters, consistent with biosynthetic gene transcriptomic activity being on average lower than most cellular processes. Across Angelobacter-SRVP2 and Angelobacter-SRVP3, BGC gene expression as a percent of all gene expression was observed to be highest in samples from 5 cm (Fig S3). A Type III PKS squalene-hopene cyclase, methyltransferases, and nonribosomal peptide synthetases were among the genes in BGCs with detectable levels of expression. Many of the genes with the highest levels of transcriptional activity in BGCs were transporter genes, including MacB-tripartite pumps and an AcrD multi-drug efflux system. These data are consistent with *Ca*. Angelobacter transcriptional activity in situ, with activity highest at shallower soil depths, and changes in transcriptional activity between samples (Fig. 4), similar to the kind previously reported in the previous metatranscriptomic experiment performed on soils with the originally reported species *Ca*. Angelobacter Gp1-AA17 [11].

## Discussion

This research expanded upon a prior finding that the genus *Ca*. Angelobacter is genomically enriched in biosynthetic gene content. While metagenome-derived genomes from this lineage have been reported before, here we expand sampling and provide evidence that this is a general feature of species in this genus. The results drew upon data from a very wide diversity of soil types, ranging from relatively dry soils that experience a Mediterranean climate (Angelo Reserve, with little or no rainfall for ~5 months per year) to permanently wet soils of a vernal pool. *Ca*. Angelobacter genomes were also identified in publicly available metagenomic datasets from agricultural soil, forest soils, and soil amended with pyrogenic organic matter. The gene inventories of these Acidobacteria may confer the metabolic flexibility needed to proliferate over a range of soil conditions. Given their ability to respire and ferment complex organic carbon compounds and their abundance and activity in the vernal pool ecosystem studied, *Ca*. Angelobacter may also contribute to carbon compound turnover in saturated, organic carbon-rich soil. In particular, the samples analyzed in our study were from fairly deep soils (20 cm and deeper), which are likely to be relatively anaerobic. Although we did not obtain DNA samples from shallower soils, we did obtain two metatranscriptome samples from a soil depth of 5 cm in which RNA expression was detected for two *Ca*. Angelobacter species, indicating that these species are likely also active in shallow soils.

Intriguingly, these specialized metabolite producers were much more abundant in the soils from this study than bacteria commonly cultivated from soil and known for their ability to produce specialized metabolites. For example, we only recovered marker genes from *Streptomyces* or *Pseudomonas* at low abundances in this study. We also demonstrate that *Ca*. Angelobacter genomes reconstructed in this study encode for as many, and sometimes more, biosynthetic genes than the originally reported genome. Further, the majority of these BGCs are unrelated, indicating substantial biosynthetic gene diversity within the genus. While functional prediction of highly divergent BGCs is challenging, we identified *Ca*. Angelobacter BGCs containing MacB tripartite efflux pumps, yet none with siderophore-specific TonB-dependent receptors. This could indicate that many of these BGCs are involved in direct interbacterial competition rather than iron acquisition.

While genome-resolved metagenomics presents its own biases as a window into soil microbial community composition - particularly sequencing bias against high GC% genes, poor DNA extraction from spores, and difficult assembly of high strain complexity species - the findings of this study imply that as yet uncultivated Acidobacteria may play an underappreciated role in chemical ecology in soil ecosystems.

## Supplementary information

Supplementary Tables

Supplementary Figure S1

Supplementary Figure S1

Supplementary Figure S3

Supplementary Figure Legends

## Data Availability

The Acidobacteria genomes and raw sequencing reads for this study will be made available under NCBI BioProject number PRJNA728365. Scripts for calculating transcriptomic gene counts, and all genomes and biosynthetic gene clusters are also made available at https://figshare.com/projects/A_widely_distributed_genus_of_soil_Acidobacteria_genomically_enriched_in_biosynthetic_gene_clusters/113286.
