# Supplementary figures and images for: A widely distributed genus of soil Acidobacteria genomically enriched in biosynthetic gene clusters
Source: ISME Commun. 2022 Aug 13;2:70. doi: 10.1038/s43705-022-00140-5 (PMC9723581; doi:10.1038/s43705-022-00140-5)

# ANI

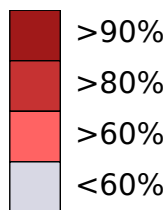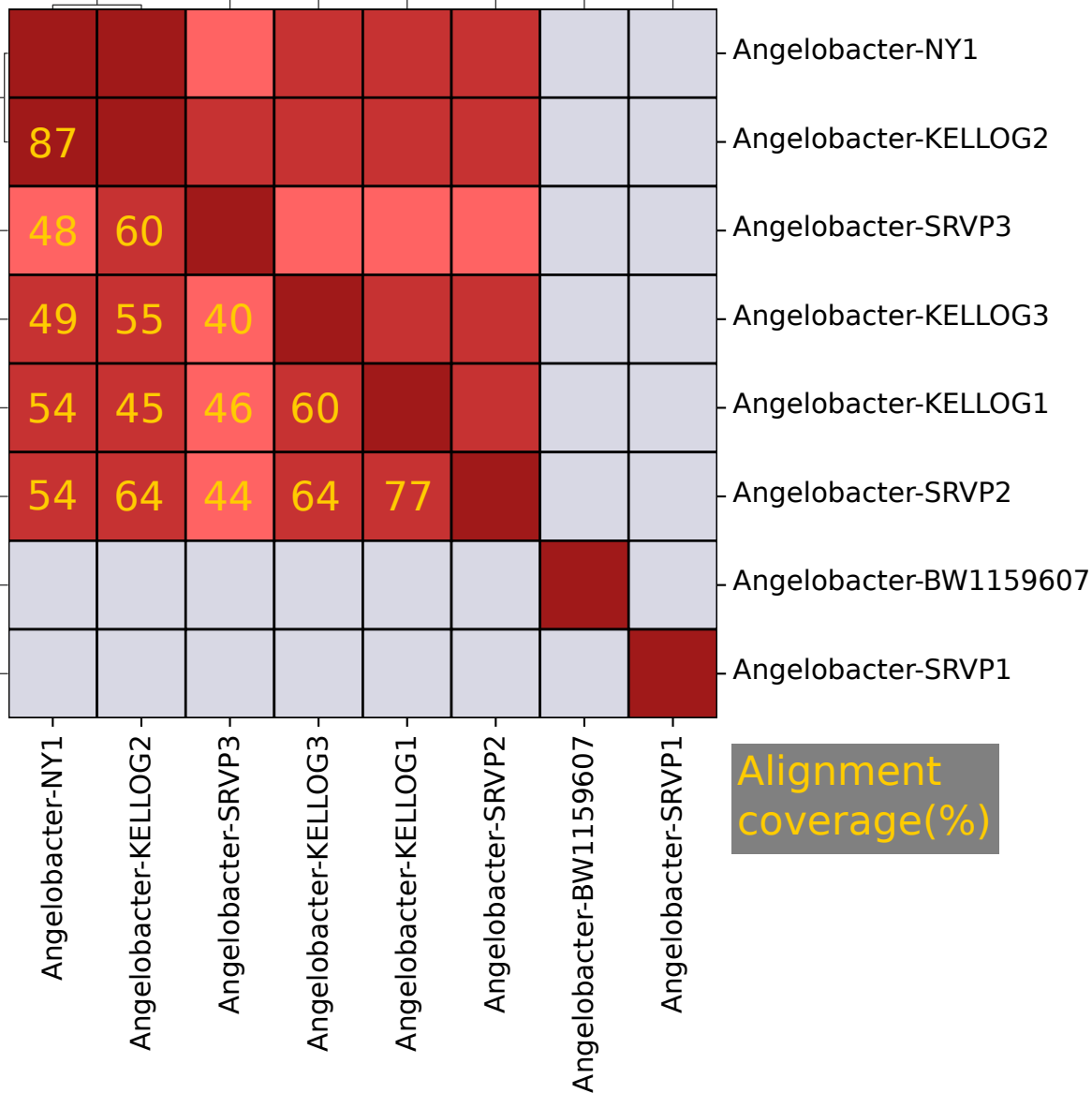

Alignment  
coverage(%)

Supplement: Supplementary file 2 — Supplementary Figure S1 [file 43705_2022_140_MOESM2_ESM.pdf]

Number of KS/CD Domains

0

50

100

150

200

250

Number of BGCS

0

20

40

60

80

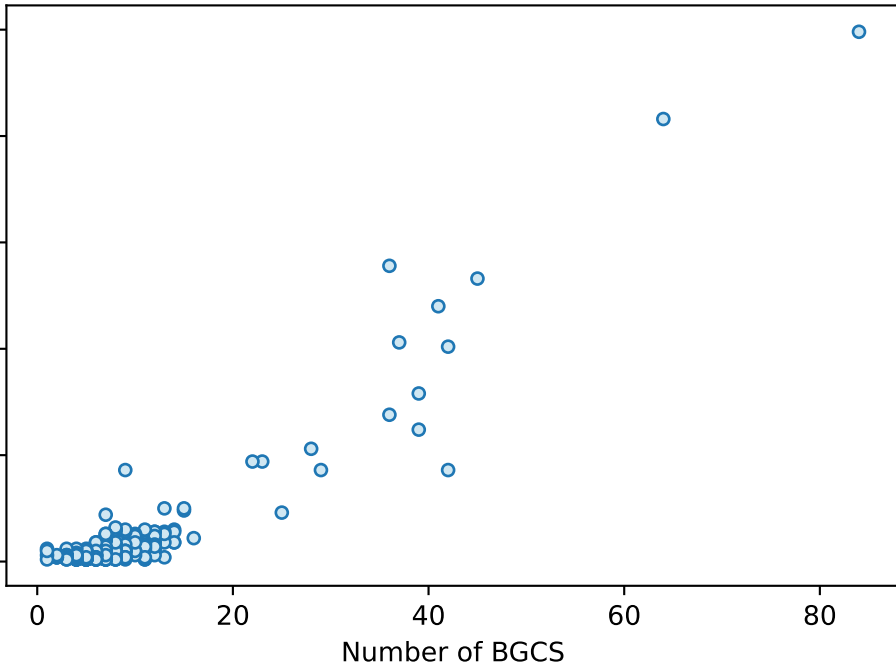

Supplement: Supplementary file 3 — Supplementary Figure S1 [file 43705_2022_140_MOESM3_ESM.pdf]

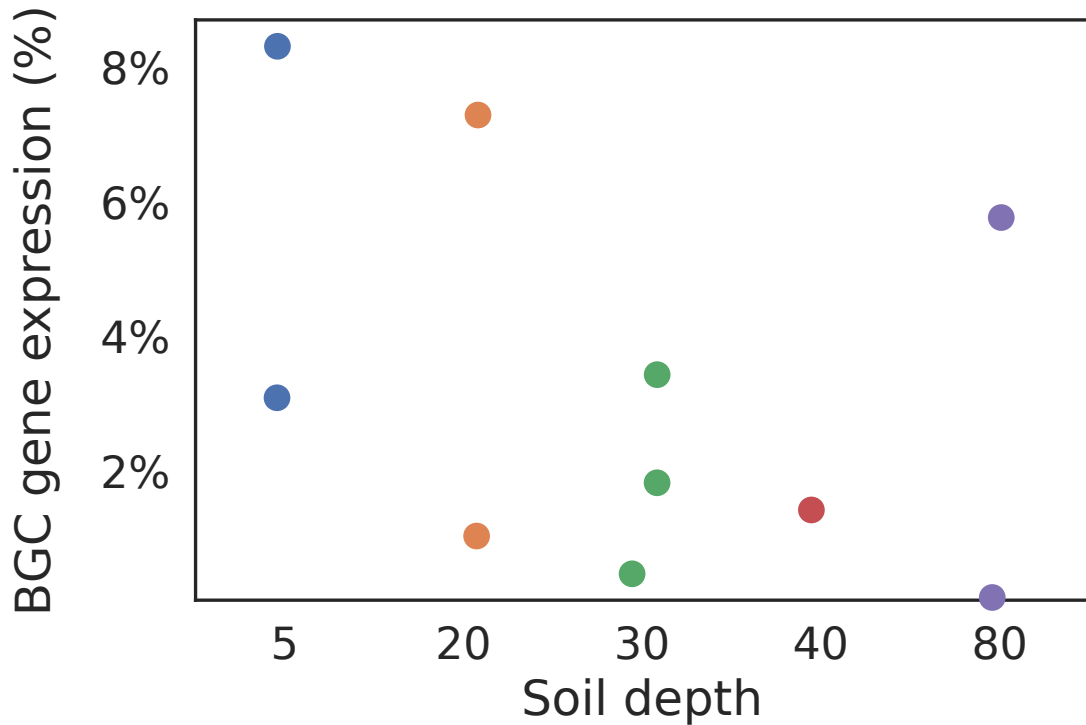

Supplement: Supplementary file 4 — Supplementary Figure S3 [file 43705_2022_140_MOESM4_ESM.pdf]
