# Supplementary material for: A widely distributed genus of soil Acidobacteria genomically enriched in biosynthetic gene clusters
Source: ISME Commun. 2022 Aug 13;2:70. doi: 10.1038/s43705-022-00140-5 (PMC9723581; doi:10.1038/s43705-022-00140-5)
Supplement: Supplementary file 5 — Supplementary Figure Legends [file 43705_2022_140_MOESM5_ESM.docx]

**Supplementary Figure Legends**

**Supplementary Figure S1**: Average Nucleotide Identity (ANI) between *Candidatus* Angelobacter genomes. ANI was determined using FastANI. The alignment coverage (genomic fraction of the alignment used to determine ANI) of each comparison is shown in each box.

**Supplementary Figure S2**: Number of BGCs per genome vs number of KS/CD domains in BGCs across all Acidobacterial genomes. Each point is an Acidobacterial genome, and the number of BGCs identified by antiSMASH in that genome is plotted on the x-axis, while the number of ketoacyl synthase and condensation domains identified within those respective BGCs is shown on the y-axis.

**Supplementary Figure S3**: BGC gene expression as a percentage of total *Candidatus* Angelobacter gene expression for each sample analyzed in this study.
